# Hyperlipoproteinaemia(a) – apheresis and emerging therapies

**DOI:** 10.1007/s11789-017-0083-2

**Published:** 2017-02-09

**Authors:** Anja Vogt

**Affiliations:** Medizinische Klinik und Poliklinik IV, Klinikum der Universität München, Ziemssenstrasse 1, 80336 München, Germany

**Keywords:** Lipoprotein(a), LDL-cholesterol, Apheresis, Atherosclerosis, New lipid-lowering therapies, Acute coronary syndrome

## Abstract

A high level of lipoprotein(a) (Lp(a)) is recognized as an independent and additional cardiovascular risk factor contributing to the risk of early onset and progressive course of cardiovascular disease (CVD). All lipid lowering medications in use mainly lower low density lipoprotein-cholesterol (LDL-c) with no or limited effect on levels of Lp(a). Niacin, the only component lowering Lp(a), is firstly often poorly tolerated and secondly not available anymore in many countries. A level of <50 mg/dl was recommended recently as the cut off level for clinical use and decision making. Since lipoprotein apheresis (LA) lowers not only LDL-c but also Lp(a) significantly, its use is recommended in some countries in very high-risk patients with early or progressive CVD. Retrospective analyses show that regular LA improves the course of CVD. This is supported by a recent prospective observational trial and data of the German Lipoprotein Apheresis Registry. Despite many treatment options, all too often it is not possible to reduce LDL-c levels to target and to reduce Lp(a) levels sustainably at all. Therefore, new drug therapies are awaited. Some of the lipid modifying drugs in development lower Lp(a) to some extent in addition to LDL-c; the only specific approach is the apoprotein(a) antisense oligonucleotide. Currently LA is the standard of care as a last resort treatment in high-risk patients with elevated Lp(a) and severe CVD despite optimal control of all other cardiovascular risk factors.

## Introduction

Lipoprotein(a) (Lp(a)) in man was first described in 1963 by Berg, who stated that the level of Lp(a) is mainly inherited and that high levels of Lp(a) are associated with premature atherosclerosis [1]. This has been confirmed thereafter by observational data and supported in recent years by genetic data establishing Lp(a) as a causal factor for the development of atherosclerosis [2–8]. The European Guidelines on vascular disease prevention in clinical practice [9] mention high levels of Lp(a) as being associated with an increased risk of CVD and suggest to use levels of Lp(a) for risk stratification in individuals at moderate risk or with a positive family history of early CVD. The consensus paper of the European Atherosclerosis Society (EAS) [10] offers guidance. Lp(a) should be measured e. g. in all individuals at intermediate or high risk of CVD, in case of premature CVD, familial hypercholesterolaemia, premature CVD or high Lp(a) in the family, progressive CVD despite statin therapy.

Lp(a) consists of a low density lipoprotein (LDL) particle and an additional protein called apoprotein(a) (apo(a)), linked to apoprotein B (apoB) 100 of the LDL particle via one disulfide bond. Mostly the level depends on the size of apo(a) [11] and both are negatively correlated. The different laboratory methods are not comparable and results cannot be converted. If comparing data the used methods have to be taken into account. The consensus statement of the EAS recommended the use of an isoform insensitive assay and suggested a level of <50 mg/dl as desirable [10]. Since risk increases with increasing levels of Lp(a) and interventional data are missing, there is no established threshold.

## Established therapies

### Drugs

Some data show either a decrease or an increase of Lp(a) by statins [12], but mostly Lp(a) is not affected by statins [10, 13, 14] nor by ezetimibe [15]. Nicotinic acid (niacin) reduces Lp(a) besides positive effects on LDL-c, high density lipoprotein-cholesterol (HDL-c), and triglycerides [16]; high doses (2–4 g) reduce Lp(a) significantly [17]. Whether this holds true for individuals with high levels of Lp(a) has never been shown. A meta-analysis of the beneficial effects of nicotinic acid on cardiovascular events [18] did not discriminate whether the lowered levels of Lp(a) might have contributed to the positive results or not. It has to be mentioned that these data are mainly from the pre-statin era and would have to be confirmed in cohorts treated in line with current options and guidelines. In the EAS consensus paper niacin is recommended to reduce high levels of Lp(a) [10]. Since 2013 niacin is not available in Europe. In summary, no established drug treatment option to reduce Lp(a) is available at the moment.

### Lipoprotein apheresis

Lipoprotein apheresis (LA) is in clinical use for over 30 years [19] and reduces apoB100 containing lipoproteins (namely LDL-c and Lp(a)). A single treatment reduces both by about 60–70%. The following increase is rapid [20]. For this reason, LA has to be repeated regularly and is done every week in most or every two weeks in some countries. Guidelines of several countries recommend LA in very high risk patients as a last resort therapy to lower LDL-c in addition to maximal (tolerated) lipid lowering medication. Few countries also consider high levels of Lp(a) as an indication for LA in very high risk patients [21–24].

There are no randomised prospective trials. LA has beneficial effects regarding endothelial function and myocardial perfusion in patients with high levels of Lp(a) [25]. Retrospective evaluations of clinical data and analyses of the German Lipoprotein Apheresis Registry (GLAR) show that cardiovascular events were reduced significantly after establishing regular LA [26–28]. One retrospective evaluation indicates that patients with elevated Lp(a) irrespective of the LDL-c level have a greater benefit from LA than patients with low Lp(a) and high levels of LDL-c [29]. One prospective open-label trial used atorvastatin plus the a selective Lp(a) lowering apheresis system (treatment group) and atorvastatin alone (control group). After 18 months a small but significant regression of coronary atherosclerosis was documented by angiography in the LA group [30]. The Pro(a)-LiFe-study, a non-randomised prospective observational multicentre study in high-risk patients, showed a reduction of major adverse cardiac events and of major adverse non-cardiac events after two [31] as well as after five years [32] of apheresis therapy.

Lipoprotein apheresis is well tolerated and safe in clinical use [24, 33] even for 2–3 decades of treatment. Several obstacles are opposed to a wide clinical use: it is time consuming, expensive, only offered in specialised centres, and mostly not covered by insurance companies.

## Emerging therapies

In recent years several new lipid modifying approaches mostly addressing LDL-c were investigated. Inhibitors of the cholesterol ester transfer protein (CETP) increasing HDL-c and reducing LDL-c are not approved yet. In a phase-2 trial Anazetrapib additionally lowered Lp(a) by 50% [34]. In the REALIZE trial Anazetrapib showed a substantial reduction of Lp(a) by 31.8% in patients with heterozygous familial hypercholesterolaemia (FH) [35]. TA-8995, a newer agent, lowered Lp(a) in patients with dyslipidaemia by 36.9% (5 mg) and 33.4% (10 mg) at week 12 in a phase 2 trial [36]. The REVEAL trial using Anacetrapib to be published in 2017 will show whether this lipid modifying approach is beneficial in patients with established vascular disease [37].

The apoB antisense oligonucleotide (ASO) Mipomersen is approved in the US but not in Europe [38, 39]. Besides the reduction of LDL-c via less hepatic production of very low density lipoprotein (VLDL) Lp(a) is reduced by a still unclear mechanism. In a randomized trial Mipomersen [40] reduced initially elevated levels of Lp(a) significant by 31.1%. In patients with heterozygous FH and coronary heart disease Mipomersen was added to lipid lowering therapy and reduced Lp(a) by 21.1% [41]. Thus, Mipomersen might reduce the necessity for lipoprotein apheresis in high-risk patients [42].

Lomitapide, an inhibitor of the microsomal triglyceride transfer protein (MTP), lowers LDL-c by reducing the assembly of VLDL in the liver as well of chylomicrons in the intestine. This is the only approach independent of the functionality of LDL-receptors. For this reason, the MTP-inhibitor Lomitapide significantly lowers LDL-c in homozygous FH (hoFH). Efficacy and safety in patients with hoFH were assessed in a single-arm, open-label, phase-3 study. Lp(a) was reduced by 19% at week 56 but at week 78 this was no longer seen [43]. Lomitapide is approved for hoFH.

### PCSK9-inhibitors

Like the LDL-receptor the proprotein convertase subtilisin/kexin type 9 (PCSK9) is produced in the liver cells and released into the circulation. If PCSK9 is attached to the complex of LDL-receptor and LDL-c the receptor is degraded after internalisation and thus cannot be recycled for further uptake of LDL-c. Loss of function mutations come along with lower levels of LDL-c lifelong and are linked to a lower rate of cardiovascular events [44]. Two PCSK9-inhibitors (fully humanized monoclonal antibodies) to mimic these beneficial “natural” effects by binding PCSK9 and thus hampering the intracellular lysis of the LDL-receptor are approved. Overall, safety and tolerability profiles are very promising in both of the huge trial programmes. Long-term results show a sustained significant reduction of LDL-c [45, 46]. The LDL-c lowering effect is dependent on the functionality of the LDL-receptors and can vary widely. Mean LDL-c reduction is about 50–60% and in some trials a lowering of Lp(a) [45–48] was seen as well (Evolocumab 25.5% [46], Alirocumab 30.2% [45]).

A pooled analysis [48] of three phase-2 Alirocumab trials specifically analysed the effect on Lp(a). Baseline levels between 2 and 181 mg/dl were parted in two groups (≤ and ≥50 mg/dl). In absolute values Lp(a) was reduced by 3.5 mg/dl and 27 mg/dl (mean), respectively, or relatively by 36% and 27% (median), respectively. In patients with LDL-receptor negative hoFH evolocumab was, as expected, ineffective in reducing LDL-c but all the same Lp(a) was lowered by 20% [49]. The upregulation of other receptors, e. g. the VLDL-receptor that mediates the uptake of Lp(a) into macrophages, might be an explanation for this finding. Some data show an increased catabolic fraction rate, others a reduced synthesis [50]. The development of bococizumab was terminated in November 2016 due to an unexpected decline of effectivity over time, a higher level of immunogenicity, and more frequent injection site reactions [51].

The results of the endpoint trials are expected in early 2017 (Evolocumab [52]) and in 2018 (Alirocumab [53]). Whether lowering Lp(a) by PCSK9-inhibitors contributes to the expected beneficial cardiovascular effects will have to be addressed in specifically designed trials.

### Apo(a) antisense oligonucleotide

The ASO molecule IONIS-APO(a)-Rx specifically addresses the mRNA of apo(a). After a positive trial in transgenic mice [54] a phase-1 trial in healthy volunteers with Lp(a) levels ≥25 nmol/l was conducted. Various doses of the ASO lowered Lp(a) significantly (no relevant reduction of other lipoproteins). The highest dose of 300 mg reduced Lp(a) by 77.8%. This effect was sustained and Lp(a) was still lowered 84 days after the last dose [55]. Data of a phase-2 trial are awaited. If effectivity and safety are shown this ASO would for the first time allow trials to address the question if the isolated lowering of high levels of Lp(a) results in lesser CVD.

## Conclusion

The new drug developments will enable us to reduce LDL-c as significantly as never before with more high-risk patients reaching their treatment goals. The results of the endpoint trials will show to what extent this contributes to the expected reduction of cardiovascular events and deaths. The question if the concomitant lowering effect of Lp(a) is beneficial in addition has never been addressed so far. This will have to be evaluated in trials specifically addressing patients with high levels of Lp(a) and CVD. It is far more reasonable to address this question with the apo(a) ASO. So far, there are no data for any established or newly developed agent proving that the Lp(a) lowering effect is beneficial in high-risk cardiovascular patients.

All available data regarding lipoprotein apheresis, though not from RCTs, at least strongly support its beneficial effect of reducing cardiovascular events. In patients with controlled cardiovascular risk factors, LDL-c at goal, progressive cardiovascular disease, and markedly elevated levels of Lp(a) lipoprotein apheresis remains the only available and optimal care. As of today, it should be considered in high-risk patients wherever it is available.
